# Changes in Carbohydrate Content and Membrane Stability of Two Ecotypes of *Calamagrostis arundinacea* Growing at Different Elevations in the Drawdown Zone of the Three Gorges Reservoir

**DOI:** 10.1371/journal.pone.0091394

**Published:** 2014-03-07

**Authors:** Shutong Lei, Bo Zeng, Zhi Yuan, Xiaolei Su

**Affiliations:** 1 Managing Office for National Assets and Laboratory Equipments, Linyi University, Linyi, China; 2 Key Laboratory of Eco-Environments in Three Gorges Reservoir Region (Ministry of Education), Chongqing Key Laboratory of Plant Ecology and Resources in Three Gorges Reservoir Region, School of Life Science, Southwest University, Chongqing, China; Beijing Forestry University, China

## Abstract

**Background:**

The Three Gorges project has caused many ecosystem problems. Ecological restoration using readily-available plants is an effective way of mitigating environmental impacts. Two perennial submergence-tolerant ecotypes of *Calamagrostis arundinacea* were planted in an experimental field in the drawdown zone. Responses of the two plant ecotypes to flooding stress in the drawdown zone were unknown.

**Methodology/Principal Findings:**

Carbohydrate content and membrane stability, two key factors for survival of plants under flooding stress, of two ecotypes (designated “dwarf” and “green”) of *C. arundinacea* growing at different elevations of the drawdown zone were investigated. Live stems (LS) and dead stems (DS) of the two plant ecotypes at eight elevations (175, 170, 162, 160, 158, 155, 152 m and 149 m) were sampled. Contents of soluble sugar, starch and malondialdehyde (MDA), as well as plasma membrane permeability of live stems were measured. The lowest elevations for survival of dwarf and green *C. arundinacea* were 160 m and 158 m, respectively. Soluble sugar content of live stems of both ecotypes decreased with elevation, with amounts from an elevation of 170 m being lower than from an elevation of 175 m. MDA content and plasma membrane permeability in live stems of green *C. arundinacea* did not increase with the decrease in elevation, while these measures in dwarf *C. arundinacea* from an elevation of 162 m were significantly higher than from an elevation of 175 m.

**Conclusions:**

Carbohydrate content, especially soluble sugar content, in both ecotypes was more sensitive to flooding stress than membrane stability. Green *C. arundinacea* had a higher tolerance to submergence than dwarf *C. arundinacea*, and thus green *C. arundinacea* can be planted at lower elevations than dwarf *C. arundinacea*.

## Introduction

Since the completion of the Three Gorges project, the water level of the reservoir fluctuates regularly from 145 m to 175 m in elevation. In October, the water level rises gradually to 175 m. By the following January, the water level starts to fall, finally dropping to 145 m in June. Thus, two water-level drawdown zones with a maximum drop of 30 m are formed along the banks of the Yangtze River, an area known as the drawdown zone of the Three Gorges Reservoir [1]. In the drawdown zone, plants suffer serial submergence stress with durations as long as 210 days at depths of up to 30 m [2]. Few plant species can tolerate such submergence stress. Therefore, the drawdown zone of the Three Georges Reservoir has become a disturbed ecosystem, leading to environmental problems such as soil erosion, pollution, and landscape damage [3], [4].

To mitigate environmental impacts, researchers have performed numerous studies [5], [6], [7], [8]. Ecological restoration using plants is an ideal option for mitigating environmental impacts in the drawdown zone of the Three Gorges Reservoir [9], [10]. At present, there is an interest in finding submergence-tolerant plants for use in ecological restoration [2], [11], [12] and in understanding the mechanisms of submergence tolerance [13], [14], [15]. Some plant species have been selected for ecological restoration in the drawdown zone of the Three Gorges Reservoir [2], [10], [11]. “Dwarf” and “green” *Calamagrostis arundinacea* are two ecotypes of a perennial grass with the ability to tolerate submergence and to subsequently recover after a period of submergence. These two plant ecotypes reproduce asexually through stem cuttings or tillers. They were planted in an experimental field in the drawdown zone for the purpose of ecological restoration. However, responses of the two ecotypes of *C. arundinacea* to flooding stress in the drawdown zone were unknown.

Generally, high amounts of carbohydrate storage and stable membrane systems can allow plants to survive under submerged conditions; plants will die if carbohydrates are exhausted or the membrane system is damaged [16], [17], [18]. Therefore, changes in carbohydrate and membrane stability of dwarf and green *C. arundinacea* stems under different elevations should be investigated for effective restoration in the drawdown zone of the Three Gorges Reservoir.

In this study, stems of dwarf and green *C. arundinacea* growing at different elevations (175, 170, 162, 160, 158, 155, 152 m and 149 m) in the drawdown zone of the Three Gorges Reservoir were collected. Changes in contents of soluble sugar, starch, malondialdehyde (MDA) in live stems (LS) and dead stems (DS), as well as plasma membrane permeability of live stems, were investigated.

## Materials and Methods

### Plant Materials

“Dwarf” and “green” *C. arundinacea* are two submergence-tolerant ecotypes of *C. arundinacea*, which were selected and cultivated by the Key Laboratory of Eco-Environments in the Three Gorges Reservoir Region (Ministry of Education) at Southwest University. The two plant ecotypes were planted into an experimental field in April 2008. The experimental field was located in the drawdown zone of the Three Gorges Reservoir with permits from the Chongqing Environmental Protection Agency. The two plant ecotypes of *C. arundinacea* are not endangered or protected species.

### Elevation Sampling

Sampling elevations were determined by the terrain of the experimental field, as well as the planting locations of dwarf and green *C. arundinacea*. In 2008, the two plant ecotypes were planted into the experimental field between 175 m and 149 m in elevation; plants survived to ∼150 m in elevation after an impoundment period of the Three Gorges Reservoir. Therefore, the sampled elevations that were used are as follows: 175, 170, 162, 160, 158, 155, 152 m and 149 m. The longest durations and the largest depths of submergence for the sampled elevations are shown in Table 1.

**Table 1:** The longest submergence durations and the largest depth of submergence for the experimental elevations, with the sampling dates.

Elevation (m)	175	170	162	160	158	155	152	149
The longest submergence durations (d)	0	124	219	246	261	289	301	337
The largest depth of submergence (m)	0	5	13	15	17	20	23	26
The sampling dates (2011)	Mar. 25	Mar. 25	Apr. 11	Apr. 26	Apr. 30	May 14	May 31	Jun. 8

### Sampling

Sampling was conducted just after the recession of water from each elevation from March to June 2011 (Table 1). Samples for investigating plasma membrane permeability were collected from the complete plant in soil, while samples for other measures were collected from live stems and dead stems. For live stems, three to six quadrats (0.5 m×0.5 m) were chosen in areas where plants survived. Ten to thirty plants with only one stem were collected in each quadrat for a replication.

For dead stems, plants with both live parts and dead parts were chosen for sampling at each elevation. Plants were randomly selected, and 15 cm of the stem above the joint of the live stem and the dead stem were clipped (4 g fresh weight per replicate). Two grams (dry weight) of dead stems were used per replication. If all plants were dead at a certain elevation, the base of the dead stem (15 cm from the land surface) was collected.

After collection and cleaning, live samples were taken back to the laboratory. Samples were placed into ultra-low temperature freezers (−80°C) for long-term storage. In addition, after cleaning and air-drying, dead stem samples were also taken back to the laboratory and placed into ultra-low temperature freezers (−80°C) for long-term storage as well.

Before analysis, samples were dried at 60°C for 72 h, and then were ground to powder (∼80 mesh) by a MM 200 grinding instrument.

### Measurement Methods

Total soluble sugar and starch contents were determined by the Anthrone-sulphuric acid method [19]. Soluble sugar was extracted from a 0.0500 g sample with 80% (v/v) aqueous ethanol in a water bath at 80°C for 40 min. The extracted solution was diluted to 50 mL after extracting 3 times. One milliliter of the extract obtained was mixed with 5 mL of an anthrone-sulphuric acid reagent described by [19]. The absorbance was measured at 627 nm using a SHIMADZU UV-2550 uv-visible spectrophotometer. Starch was converted to soluble sugar by using 6 mol/L HCl. Starch content was calculated by multiplying by the coefficient of hydrolysis (0.9).

Malondialdehyde content was determined by the glucosinolate barbituric acid method described by Heath and Packer (1965) [20]. A pre-weighed sample (0.1000 g) was extracted with 2 mL of a potassium phosphate buffer (50 mmol/L, pH 7.8), and diluted to 10 mL with the above buffer. The reaction mixture contained 2 mL of the sample supernatant and 2 mL of TBA (0.5%, w/v). The reaction was incubated in a boiling water bath for 10 min, and then was cooled in a cold water bath. The cooled mixture was centrifuged at 5000 r/min at 4°C for 20 min. The absorbance of the supernatant layer was measured with a SHIMADZU UV-2550 uv-visible spectrophotometer at three wavelengths: 532, 600 and 450 nm. The MDA content was expressed as nmol/g DW.

Plasma membrane permeability was determined via the conductivity method [19]. Live stems were cleaned carefully with distilled deionized water (DDW) and were cut into segments of 0.8 cm in length. Ten segments were placed in glass tubes containing 15 mL of DDW. The tubes were air-exhausted in a vacuum drier for 40 min and were incubated in a water bath at 25°C for 4 h and the initial electrical conductivity (L_1_) in the medium was measured. The samples were boiled at 100°C for 40 min to release all electrolytes, cooled and the final electrical conductivity (L_2_) was measured. Plasma membrane permeability was calculated by (L_1_/L_2_)×100%.

### Statistical Analysis

One-way analysis of variance (ANOVA, SPSS 13.0) was used for testing the effects of submergence on carbohydrate content and membrane stability. Duncan’s multiple test was used to test if carbohydrate content and membrane stability differed between elevations. A t-test was used for testing differences between measures of lives stems and dead stems.

## Results

The lowest elevations at which dwarf and green *C. arundinacea* could survive were 160 m and 158 m, respectively. At the lowest elevations, there were only underground parts living for dwarf *C. arundinacea*, while a few short live stems were found for green *C. arundinacea*. At elevations below the lowest elevations, there were only completely dead plants (above 155 m in elevation) or missing plants (below 155 m in elevation).

### Soluble Sugar Content

**Figure 1:**
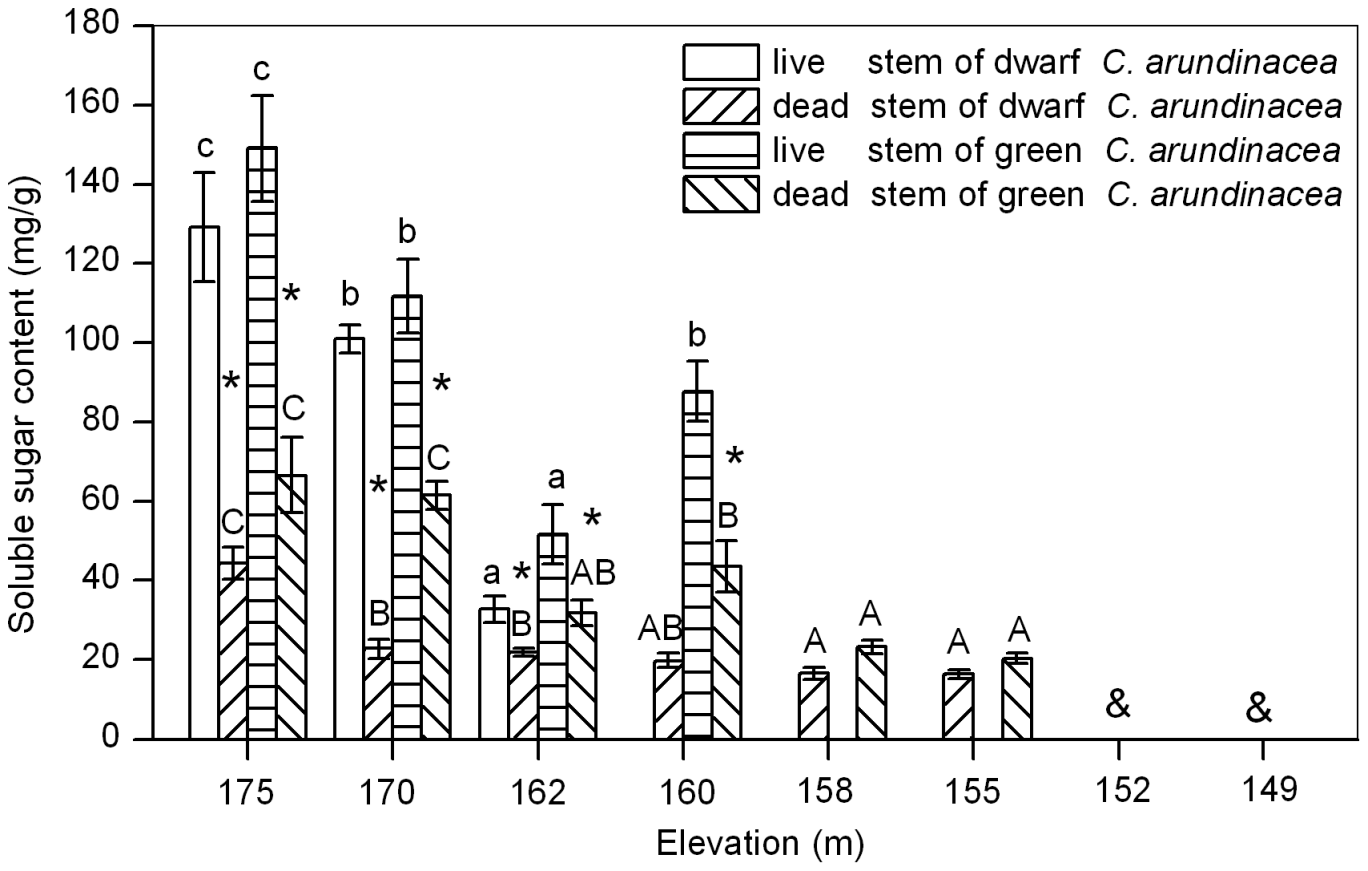
Effects of flooding on soluble sugar content in live stems (LS) and dead stems (DS) of dwarf and green *Calamagrostis arundinacea*. Values are mean ± SE based on 3–6 independent assays. Different letters indicate statistical significance (*P*<0.05) according to Duncan’s test, with lowercase for LS and capitals for DS. * indicates that a significant difference occurred between soluble sugar content of LS and DS according to T-test, while & indicates that no samples were available at the elevation.

Carbohydrate content of live stems were affected by submergence and there were differences in soluble sugar content of live stems from different elevations (ANOVA, dwarf *C. arundinacea*, *P*<0.001; green *C. arundinacea*, *P*<0.001; Fig. 1). Soluble sugar content of dwarf and green *C. arundinacea* live stems decreased with elevation. Soluble sugar content of live stems of both plant ecotypes from an elevation of 162 m were less than 50% of plants from an elevation of 175 m (dwarf *C. arundinacea*, 25.4%; green *C. arundinacea*, 34.7% ). There were differences between soluble sugar content of live and dead stems, with dead stems being significantly lower than live stems at an elevation of 175 m and at submerged elevations.

### Starch Content

**Figure 2:**
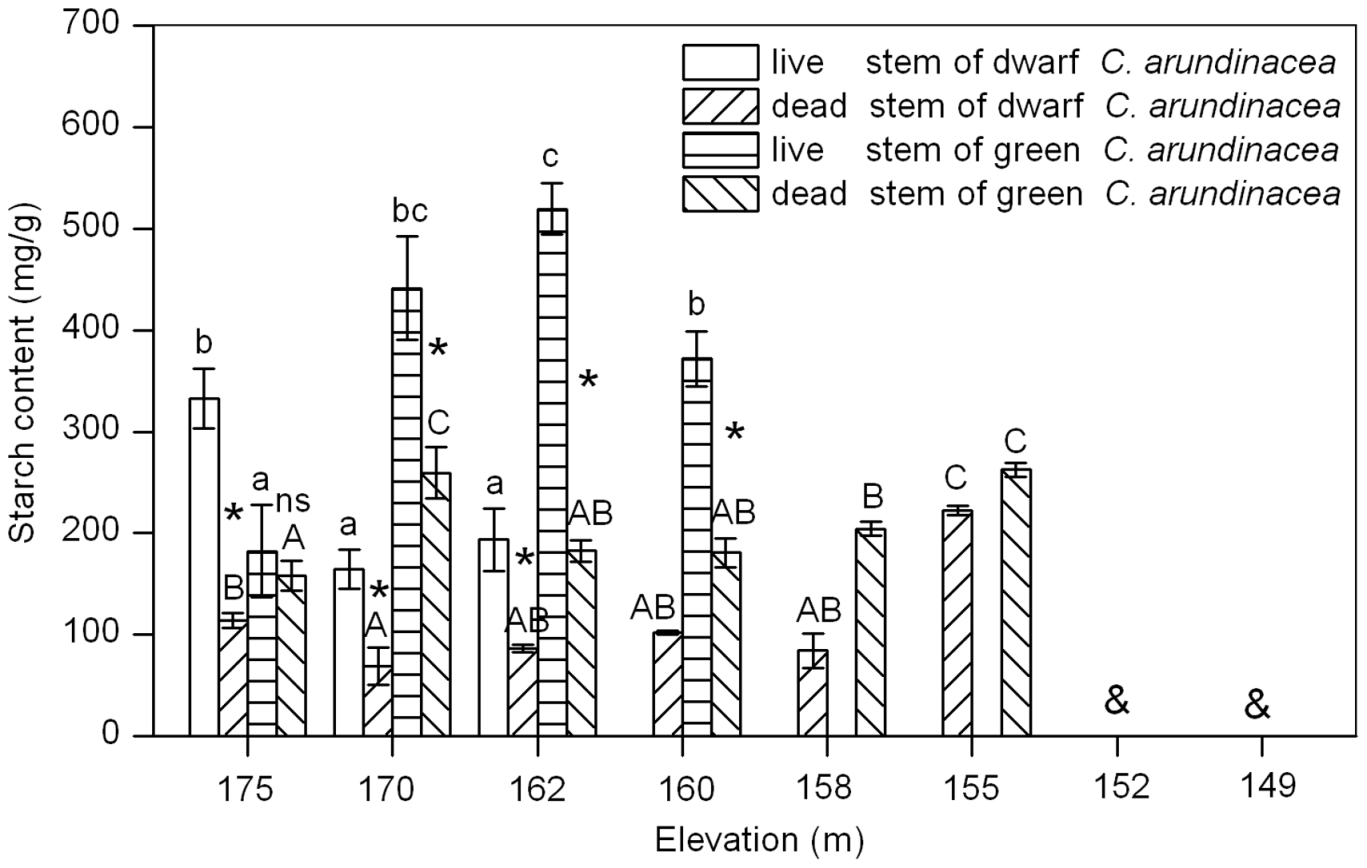
Effects of flooding on starch content in live stems (LS) and dead stems (DS) of dwarf and green *C. arundinacea*. Values are mean ± SE based on 3–6 independent assays. Different letters indicate statistical significance (*P*<0.05) according to Duncan’s test, with lowercase for LS and capitals for DS. ns indicates that no significant differences occurred between starch content of LS and DS, while * indicates that a significant difference occurred between starch content of LS and DS according to T-test and & indicates that no samples were available at the elevation.

Starch content of live stems were affected by submergence and there were significant differences between starch content of live stems from different elevations (ANOVA, dwarf *C. arundinacea*, *P* = 0.0012; green *C. arundinacea*, *P*<0.001; Fig. 2). Starch content of dwarf *C. arundinacea* live stems decreased with elevation, with starch content in plants from 170 m and 162 m elevation being significantly lower than starch content in plants from an elevation of 175 m. Starch content of green *C. arundinacea* live stems increased then decreased as elevations decreased, with peak starch content in plants at 162 m. Starch content of dead stems of both species was significantly lower than starch content of live stems from submerged elevations. No significant difference was found between starch content of green *C. arundinacea* live stems and dead stems from an elevation of 175 m; starch content of dwarf *C. arundinacea* dead stems was significantly lower than live stems from an elevation of 175 m.

### Plasma Membrane Permeability of Live Stems

**Figure 3:**
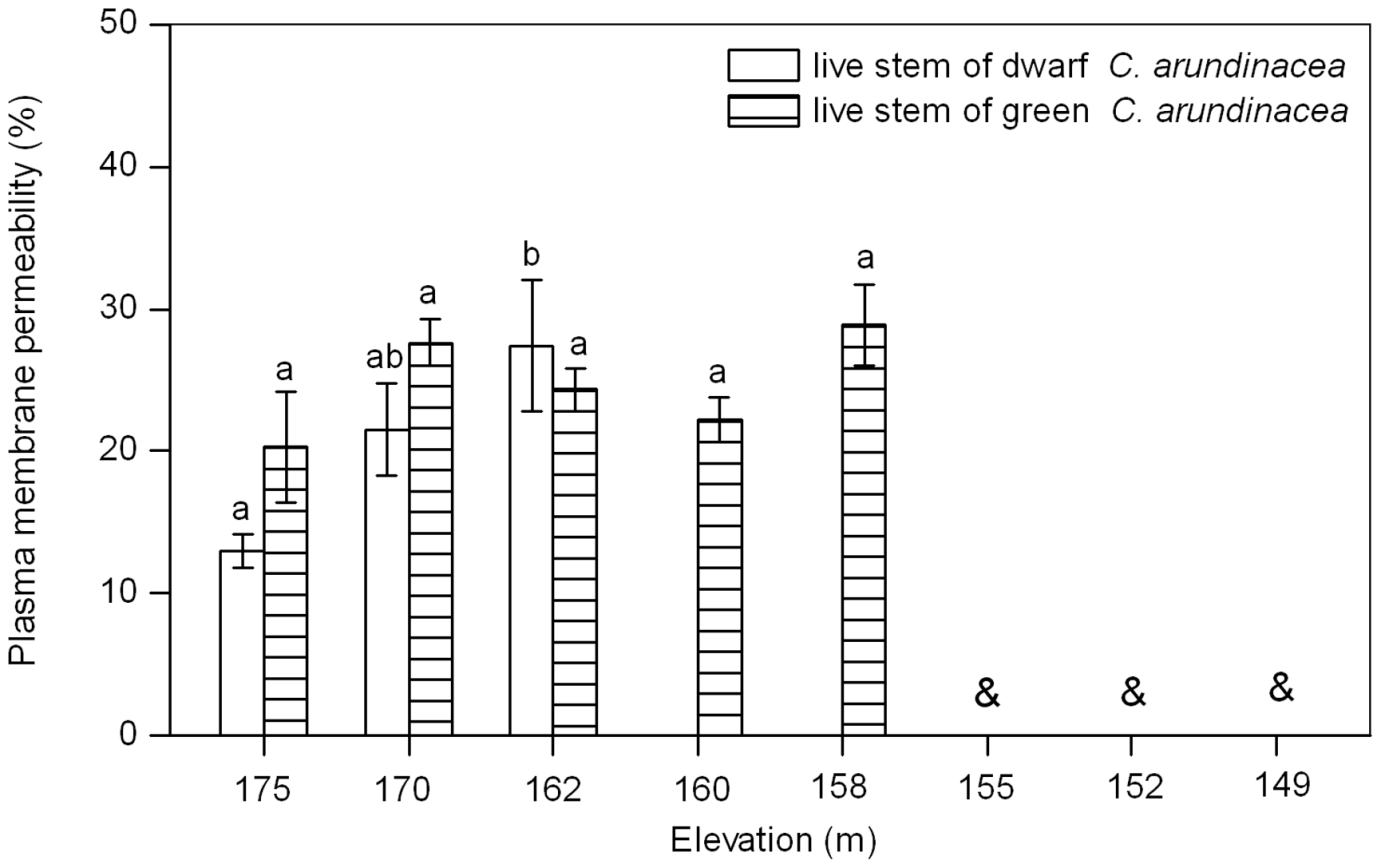
Effects of flooding on plasma membrane permeability of dwarf and green *C. arundinacea*. Values are mean ± SE based on five independent assays. Different letters indicate statistical significance (*P*<0.05) according to Duncan’s test, while & indicate that no samples were available at the elevation.

Membrane integrity of dwarf *C. arundinacea* was affected by submergence and the degree of membrane damage (plasma membrane permeability) increased significantly with a decrease in elevation (ANOVA, *P* = 0.0304; Fig. 3). Membrane permeability of dwarf *C. arundinacea* from an elevation of 162 m was significantly higher than membrane permeability of plants from an elevation of 175 m, but it was still lower than 30%. Membrane stability of green *C. arundinacea* was not affected by submergence and no significant difference was found between membrane permeability of plants from different elevations (ANOVA, *P* = 0.1987; Fig. 3).

### MDA Content

**Figure 4:**
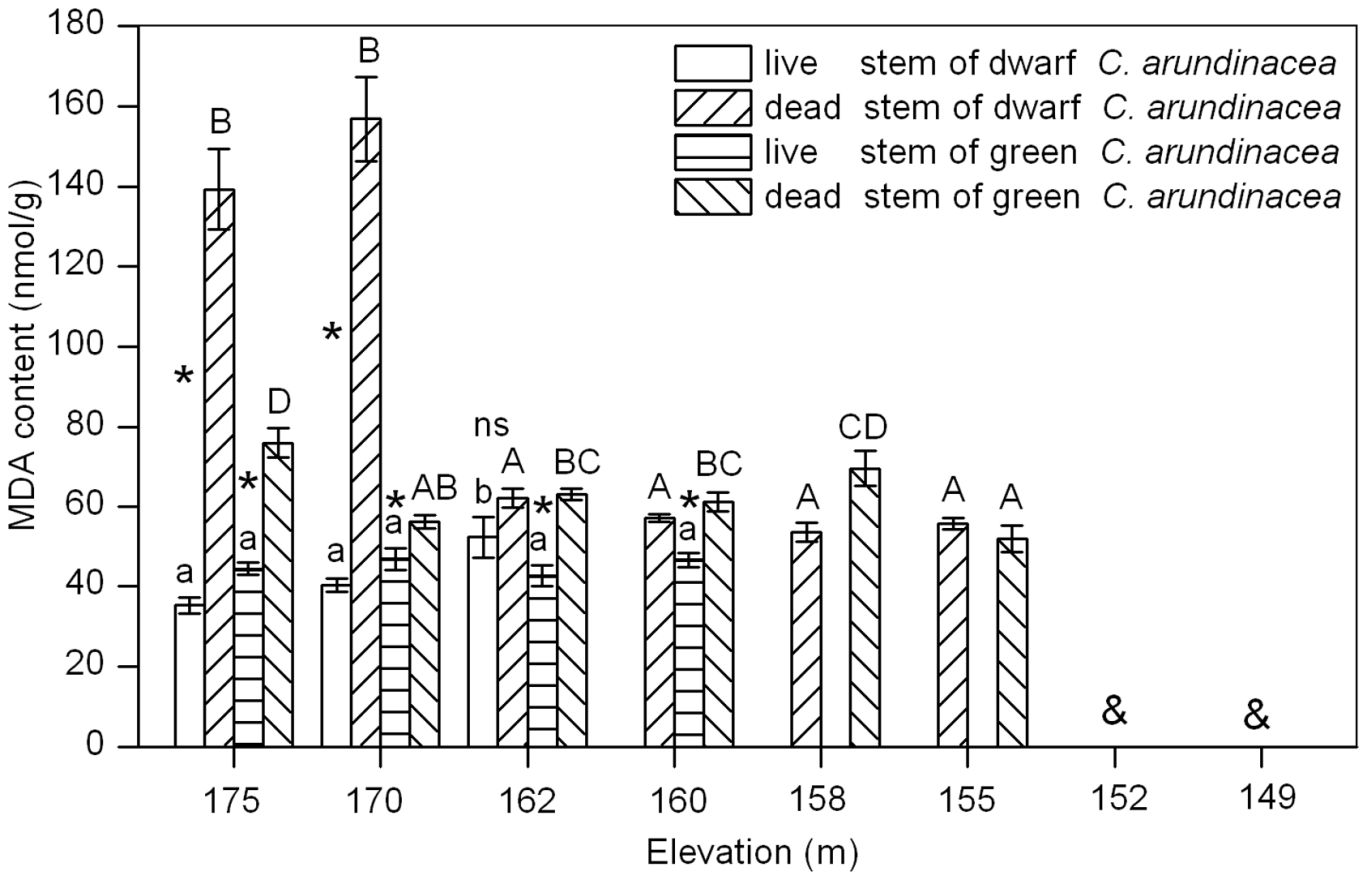
Effects of flooding on MDA content in live stems (LS) and dead stems (DS) of dwarf and green *C. arundinacea*. Values are mean ± SE based on six independent assays. Different letters indicate statistical significance (*P*<0.05) according to Duncan’s test, with lowercase for LS and capitals for DS. ns indicates that no significant differences occurred between MDA content of LS and DS, while * indicates that a significant difference occurred between MDA content of LS and DS according to T-test and & indicates that no samples were available at the elevation.

Membrane stability of dwarf *C. arundinacea* live stems were affected by submergence as evidenced by differences in MDA content of live stems from different elevations (ANOVA, *P = *0.0035; Fig. 4). MDA content of dwarf *C. arundinacea* live stems increased significantly with a decrease in elevation, with MDA content of plants from an elevation of 162 m being 48.1% higher than from an elevation of 175 m. Membrane stability of green *C. arundinacea* live stems was not affected by submergence as evidenced by the lack of significant differences between MDA content of live stems from different elevations (ANOVA, *P* = 0.5514; Fig. 4). MDA content of dead stems of dwarf and green *C. arundinacea* were significantly higher than live stems from an elevation of 175 m and from submerged elevations.

## Discussion

Non-structural carbohydrates in plant tissues mainly include soluble sugar and starch. The former can be used directly by the plant, while the latter is the carbohydrate storage of higher plants [21]. Previous studies have shown that possessing a large amount of carbohydrate reserves is a key factor in plant tolerance to long-term submergence [22], [23], [24]. It not only directly affects the survival of plants during submergence [25], but also plays an important role in the recovery growth of plants after submergence [26]. In the present study, soluble sugar content of live stems of dwarf and green *C. arundinacea* decreased with elevation; live stems of dwarf and green *C. arundinacea* from an elevation of 162 m decreased by 74.6% and 65.3%, respectively, compared to those from an elevation of 175 m (Fig. 1). This indicated that the two flooding-tolerant plants consumed large amounts of soluble sugar and high soluble sugar content was likely a key factor for plants in tolerating flooding stress. In addition, starch content of live stems of dwarf *C. arundinacea* also decreased with elevation, indicating that starch might also be a limiting factor under flooding stress. It was assumed that carbohydrate consumption resulted in the death of dwarf and green *C. arundinacea* plants below an elevation of 158 m. Carbohydrates obtained during short respites from submergence (Table 1) might be insufficient to support carbohydrate consumption of plants under flooded conditions. Therefore, when submergence-tolerant plants are to be used in ecological restoration, they must be planted into fields at elevations that enable them to recover sufficiently after submergence.

Membrane stability is important for cellular function, and only cells with stable membrane systems can maintain their normal physiological function. Therefore, membrane stability is usually used for measuring the extent of injury of plants under stress, and indices to evaluate membrane stability include lipid peroxidation (e.g., MDA, ethane) and membrane permeability (indicating membrane integrity) [21]. In the present study, MDA content of dwarf and green *C. arundinacea* dead stems were significantly higher than those of live stems at an elevation of 175 m and at submerged elevations, providing evidence that death may be related to membrane damage during submergence. However, when plants endured a complete submergence of 13 m of depth for 219 d at an elevation of 162 m (Table 1), plasma membrane permeability and MDA content of green *C. arundinacea* live stems were not significantly higher than these measures for live stems from an elevation of 175 m. MDA content of dwarf *C. arundinacea* live stems was only 48.1% higher than MDA content of plants from an elevation of 175 m, while plasma membrane permeability of these plants was lower than 30% (Fig. 3 and 4). These results indicate that a submergence-tolerant plant can maintain membrane stability to a certain extent under submerged conditions, consistent with the results of Lei et al. [27]. Therefore, it is not necessary to focus on membrane stability of these two plants when implementing ecological restoration in the drawdown zones of the Three Gorges Reservoir.

In the present study, green *C. arundinacea* live stems had higher carbohydrate storage (especially for starch) than dwarf *C. arundinacea* live stems (Fig. 1 and 2). Membrane stability of green *C. arundinacea* live stems was more stable than dwarf *C. arundinacea* live stems (Fig. 3 and 4). Green *C. arundinacea* had a better ability to tolerate submergence compared to dwarf *C. arundinacea*, as evidenced by differing survival of the two plants. Therefore, green *C. arundinacea* can be planted at lower elevations than dwarf *C. arundinacea* when the two plants are used for ecological restoration in the drawdown zones of the Three Gorges Reservoir.
